# Histological classification of Japanese IgA nephropathy with a small number of glomeruli using Bayes’ theorem

**DOI:** 10.1038/s41598-023-45734-8

**Published:** 2023-10-31

**Authors:** Takeshi Nakata, Masato Tanigawa, Akihiro Fukuda, Hirotaka Shibata

**Affiliations:** 1 Department of Endocrinology, Metabolism, Rheumatology and Nephrology, Faculty of Medicine, Oita University, 1-1 Idaigaoka, Hasama-machi, Yufu city, Oita, 879-5593 Japan; 2 Department of Biophysics, Faculty of Medicine, Oita University, Yufu-City, Japan

**Keywords:** Kidney diseases, Glomerulus, IgA nephropathy

## Abstract

In Japan an original pathological classification of IgA nephropathy was used, while Oxford classification of IgA nephropathy was used globally. The Oxford classification requires ≥ 8 glomeruli while the Japanese classification requires ≥ 10. Ninety-nine patients diagnosed with IgA nephropathy were included. To determine the accuracy of histological staging, we calculated the posterior probability using Bayes' theorem and adopted three model of prior distribution. First, the actual staging distribution was reclassified using the beta distribution (reclassified distribution). Second a model with the same distribution (actual distribution) as the actual staging was used. Third, a model assuming that all cases are equally distributed (equal distribution) was used. The median number of collected glomeruli was 12 (8–19). There were 33 cases (33%) wherein the glomerular count was ≤ 9. When only cases with ≥ 10 glomeruli were included, the median posterior probability was 91% (74–99) (actual distribution, 90% [74–98]; equal distribution, 85% [73–96]). Even among the 33 cases with ≤ 9 glomeruli, there were approximately 7 cases in which the posterior probability was ≥ 90% for each model. Using Bayesian probabilistic analysis, it was possible to evaluate the histologic classification of IgA nephropathy, even when the number of obtained glomeruli was ≤ 9.

## Introduction

IgA nephropathy is one of the most common types of primary glomerulonephritis in the world^1,2^ with a prognosis ranging from good to poor^3^. Some reports suggest that 40% of patients with IgA nephropathy reach end-stage renal disease (ESRD) within 20 years^4^. Assessing each patient’s prognosis is therefore particularly important, as some immunosuppressive therapies are known to be effective treatments for IgA nephropathy^5–9^.

To date, renal biopsy is the only way to definitively diagnose IgA nephropathy; however, the procedure may cause serious complications, including death^10^. Therefore, nephrologists pay exceptional attention to prevent adverse effects when performing renal biopsy.

Although the Oxford classification is widely used for the pathological classification of IgA nephropathy worldwide^11–14^, the Japanese Society of Nephrology published original pathological classification in Japan^8,15–18^. A notable difference between the Oxford and Japanese pathological classifications is the use of “split” versus “lumped” systems. In the Oxford classification, four components of histological features—mesangial hypercellularity: M0, M1; endocapillary hypercellularity: E0, E1; segmental sclerosis: S0, S1; crescent of the glomeruli: C0, C1 and interstitial fibrosis or tubular atrophy: T0, T1—were used to assess IgA nephropathy. Conversely, in the Japanese classification, histological classification was divided into four categories: histological Grades I, II, III, and IV. Histological grade was divided by the percentage of glomeruli with pathological variables, thereby predicting progression to ESRD^18^.

IgA nephropathy is a diffuse glomerular disease, and even a single glomerulus can be enough to confirm diagnosis^11^. However, the Oxford classification requires ≥ 8 glomeruli, whereas the Japanese classification requires ≥ 10 glomeruli per biopsy for proper classification. While it is crucial to obtain sufficient glomeruli for accurate histological classification of IgA nephropathy, this is not always possible. It is difficult to know exactly how many glomeruli can be obtained during renal biopsy; therefore, even if only a few glomeruli are obtained from a single kidney biopsy, it would be extremely useful for histopathological severity classification to be applied.in renal specimens.

Bayes’ theorem is widely used when examining the probability of an event based on prior knowledge of conditions that may be related to the event^19–21^. Using Bayesian analysis, we attempted to demonstrate the probability of a case being "truly" classified into a severity category, even if few glomeruli are collected in a single kidney biopsy.

## Methods

This was a cross-sectional study conducted at a single center at Oita University Hospital. The study was approved by the ethics committee of Oita University (No. 1615). The inclusion criteria were an age > 18 years and IgA nephropathy diagnosed by renal biopsy at Oita University Hospital between 2000 and 2009. The exclusion criterion was secondary IgA nephropathy, such as IgA vasculitis (Henoch–Schönlein purpura) or liver cirrhosis. We used the third edition of the IgA nephropathy classification created by the Japanese Society of Nephrology. Pathological variables for acute lesions were cellular crescent, tuft necrosis, and fibrocellular crescent; those for chronic lesions were global sclerosis, segmental sclerosis, and fibrous crescent. Histological grade was indicated by the percentage of glomeruli with pathological variables: Grade I was < 25%, Grade II was between ≥ 25% and < 50%, Grade III was between ≥ 50% and < 75%, and Grade IV was ≥ 75% (Table 1).

**Table 1:** Histological classification of IgA nephropathy in Japan.

Histological grade	% glomeruli with pathological variables* predicting progression to end-stage renal disease	Acute or chronic
H-grade I	0–24.9%	A or A/C or C
H-grade II	25–49.9%	A or A/C or C
H-grade III	50–74.9%	A or A/C or C
H-grade IV	> 75%	A or A/C or C

*Acute lesions (A): cellular crescent, tuft necrosis, fibrocellular crescent; Chronic lesions (C): global sclerosis, segmental sclerosis, fibrous crescent.

Certified pathologists diagnosed IgA nephropathy using light and fluorescence microscopy. We collected all cases pathologically diagnosed with IgA nephropathy, even if few glomeruli were obtained.

### Using Bayes' Theorem for Probabilistic Analysis in IgA Nephropathy

Bayes' theorem was used for probabilistic analysis. As mentioned above, Bayes' theorem can be used to determine whether the results of a test are true. In addition, when using Bayes' theorem, it is important to know what kind of information to use as a prior probability.

#### Setting up the model for the prior distribution

##### Model of prior distribution

Ninety-nine cases were histologically distributed into one of four grades as described above. The beta distribution (Table 2)—a conjugate prior in Bayesian estimation when the likelihood function is a Bernoulli or binomial distribution—was used to reclassify these cases to approximate their actual distribution.

**Table 2:** BETA distribution.

f(x) = kx^p−1^(1 − x) ^q^−^1^	
(0 < x < 1, k = B(p, q)^−1^ , p = 0.2, q = 1.2, x = 0.0625, 0.1875, 0.3125, 0.4375, 0.5625, 0.6875, 0.8125, 0.9375)	

The beta distribution—a conjugate prior in Bayesian estimation when the likelihood function is a Bernoulli or binomial distribution—was used to approximate the actual distribution of these cases.

p: shape parameter, q: shape parameter, B(p, q): beta function.

##### Prior distribution model for validation

**Figure 1:**
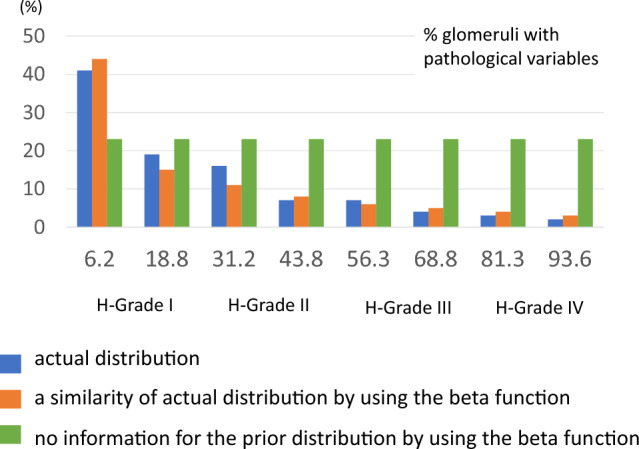
Three models of prior distribution. Actual distribution by ninety-nine cases (blue), Reclassified distribution using beta distribution (orange), and equal distribution (green). To make it easier to understand visually, a distribution graph was created with each of the four classifications divided into two in the center and eight categories (for H-Grade I 6.2 and 18.5 instead of 25, for H-Grade II 31.2 and 43.5 instead of 50, for H-Grade III 56.3 and 68.8 instead of 75, for H-Grade IV 81.3 and 93.6 instead of 100).

To examine the effect of prior distribution on the results, we used the same distribution as the actual staging and equal distributions as the prior distribution models. For equal distribution, it was assumed that the four histological severity categories (Grade 1–4) were equally distributed (noninformative prior). Uninformative prior is also used to compare other distributions in other populations when prior distribution is unknown. To make it easier to understand visually, a distribution graph was created with each of the four classifications divided into two in the center and eight categories (Fig. 1).

#### Calculation method of posterior probability

Binomial distribution was used to calculate the posterior probability of how accurately patients were classified into histological categories (H-Grades I–IV) (Table 3).

**Table 3:** Binomial distribution.

*p*_x_ = _n_C_x_ *p*^x^ (1 − *p*)^n−x^	

*P* = probability, C: binomial coefficient.

n = the number of obtained glomeruli, x = the number of glomeruli with pathological variables* predicting progression to ESRD.

Binomial distribution was used to calculate the posterior probability of how accurately patients were classified into histological categories.

### Ethical approval

This study was approved by the ethics committee of Oita University. Individual information was not collected. The study protocol adhered to the guidelines stipulated in the Helsinki Declaration and Clinical Trials Act of the Ministry of Health, Labour and Welfare in Japan. The research purpose was known to the participants before the survey. They were informed that their answers to the survey would be regarded as informed consent.

## Results

**Figure 2:**
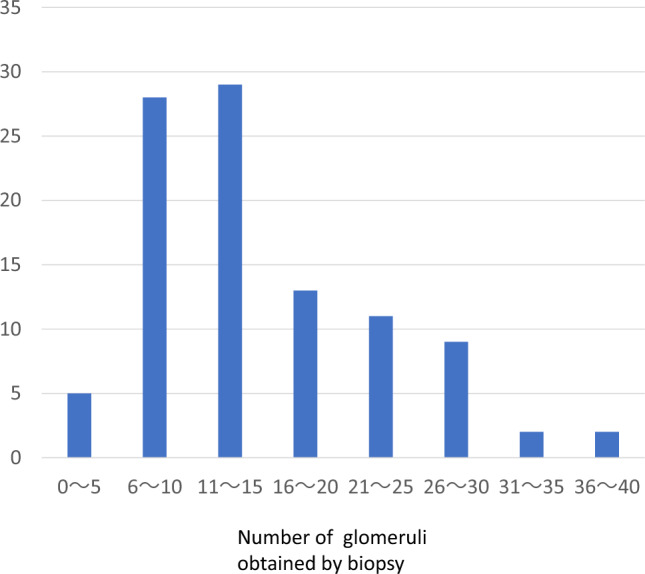
Distribution of number of glomeruli obtained by renal biopsy (all cases n = 99).

A total of 99 patients participated in the study. Characteristics of the participants are listed in Table 4. The result of the Oxford classification of IgA nephropathy (MEST-C) are shown in Table 5. The median total number of collected glomeruli was 12 (7–19). Figure 2 shows the distribution of the number of glomeruli obtained from renal biopsies.

**Table 4:** Patient characteristics.

	≦9 cases	≧10 cases	All cases
Number of cases	33	66	99
Number of glomeruli	6.70 ± 2.00, 7 [6–8]	17.5 ± 6.83, 15 [12–22]	13.9 ± 7.70,12 [8–18]
Number of total sclerotic glomerulus	1.60 ± 1.82	2.32 ± 3.27	2.08 ± 2.88
Percentage of total sclerotic glomerulus (%)	21.7 ± 24	13.4 ± 17	16.2 ± 20
Gender (male %)	42.4	53	49.5
Age ± SD	42.48 ± 12.78	37.9 ± 12.76	39.44 ± 12.88
Blood Pressure ± SD (mmHg)	124.33 ± 15.14/75.06 ± 11.53	118.49 ± 15.03/72.00 ± 11.41	120.46 ± 15.25/73.02 ± 11.49
IgA ± SD (mg/dl)	360.53 ± 134.84	348.71 ± 132.83	352.65 ± 132.93
IgA/C3	3.35 ± 1.17	3.46 ± 1.55	3.43 ± 1.43
Cr ± SD (mg/dl)	0.94 ± 0.34	0.84 ± 0.34	0.87 ± 0.38
eGFR ± SD (ml/min)	71.05 ± 27.09	80.67 ± 28.58	77.46 ± 28.32
U-TP(g /day or g/gCr)	1.07 ± 1.82	0.76 ± 0.87	0.87 ± 1.29
U-OB(− or ± , + , 2 + , 3 + %)	33.3, 15.2, 30.3, 21.2	10.9, 12.5, 34.4, 42.2	18.6, 13.4, 33.0, 35.1

U-OB: urinary occult blood.

U-TP: urinary total protein.

**Table 5:** The result of Oxford classification (MEST-C).

Score	0	1	2
M	21	78	–
S	45	54	–
E	81	18	–
T	71	26	2
C	67	30	2

M, Mesangial hypercellularity; M0, Absent to ≦ 50% of glomeruli; M1 > 50% of glomeruli; E, Endocapillary hypercellularity; E0, Absent; E1, Present; S, Segmental sclerosis/adhesions/synechiae; S0, Abesnt; S1, Present; T, Tubular atrophy and interstitial fibrosis; T0, Absent to ≦ 25% of the cortex; T1, 25–50% of the cortex; T2 > 50% of the cortex; C, Crescents; C0, Absent; C1, 1–24% of the glomeruli; C2 ≧25% of glomeruli.

Thirty-three cases (33%) wherein the numbers of obtained glomeruli were ≤ 9 were regarded as inappropriate for properly judging the pathological stage.

The posttest probabilities of actual, reclassified, and equal distributions were 86% (74–98), 85% (74–99), and 79% (73–96), respectively, for the cases with 10 or more glomeruli (Table 6).

**Table 6:** Results of three models of prior distribution.

		Analysis of cases with ≥ 10 glomeruli	Analysis of cases with ≤ 9 glomeruli	Analysis of all cases (n = 99)
	Number of cases	n = 66 (66%)	n = 33 (33%)	99
	Number of glomeruli	17.5 ± 6.83*15[12, 22] **	6.70 ± 2.00*7 [6–8] **	13.9 ± 7.70* 12 [8–18]**
Actual distribution	Posttest probability	85% ± 15* 90% [74–98]**	69% ± 22* 75%[48–90]**	79% ± 19* 86% [69–96]**
Reclassified distribution	Posttest probability	85% ± 15* 90% [74–99]**	70% ± 24* 75%[50–92]**	80% ± 20* 85% [67–97]**
Equal distribution	Posttest probability	82% ± 15* 86%[72–96]**	65% ± 17*58%[51–79]**	76% ± 17* 79% [60–93]**

*Express Mean and standard deviation.

**Express Median and Quartiles.

### Analysis of ≤ 9 glomeruli considered histologically classifiable with the same degree of accuracy as cases ≥ 10 glomeruli each three prior distributions (Table 7)

**Table 7:** Actual patterns of specimens ≤ 9 glomeruli considered histologically classifiable with the same degree of accuracy as cases ≥ 10 glomeruli.

Number of all glomeruli	Number of glomeruli with lesions	Posttest probability (%)		
		Actual distribution	Reclassified distribution	Equal distribution
2	0	81#1	84#1	58#1
3	0	86#1	89#1	69#1
4	0	90#1	92#1	77#1
5	0	92#1	94#1	83#1
6	0	94#1	96#1	87#1
7	0	96#1	97#1	90#1
8	0	97#1	98#1	93#1
9	0	98#1	98#1	95#1
9	9	90#4	91#4	95#4

^#^1 is posttest probability for Grade I #4 is posttest probability for Grade IV.

#### Actual distribution

Using the model with the actual distribution, even if the number of glomeruli collected was ≤ 9, if the total number of glomeruli with lesions was 0, the patient was classified with H-Grade I; the posttest probability could be as high as 81 to 98%, even if the numbers of glomeruli is 2 to 9 (Table 7).

#### Reclassified distribution

The result of reclassified distribution was the almost same as actual distribution (Table 7).

#### Equal distribution

The result of equal distribution was little bit lower of post probability than other distributions. Although, the judgment of the histological classification was the same as other prior distribution (Table 7).

### Analysis of ≥ 10 glomeruli considered histologically classifiable with low degree of accuracy (Table 8)

**Table 8:** Actual patterns of specimens with more than 10 glomeruli considered histologically classifiable with a low degree of accuracy.

Number of all glomeruli	Number of glomeruli with lesions	Posttest probability (%)		
		Actual distribution	Reclassified distribution	Equal distribution
10	3	54#1	50#1	61#1
10	6	56#2	56#2	63#2
11	5	65#1	63#1	57#1
13	4	60#1	57#1	67#1
15	8	49#2	49#2	58#2
19	5	52#1	48#1	61#1

^#^1 is posttest probability for Grade I.

^#^2 is posttest probability for Grade II.

#### Actual distribution

By contrast, even if the total number of glomeruli collected was ≥ 10, if the total number of glomeruli collected was 10–19 and the total number of glomeruli with lesions was 3–8, patients were classified with H-Grade II or III, and the accuracy of the classification was 49%-67%, indicating low reliability of the decision (Table 8).

#### Reclassified distribution

The result of reclassified distribution was the almost same as actual distribution (Table 8).

#### Noninformative prior distribution

The result of noninformative prior distribution was the almost same as the other distributions (Table 8).

## Discussion

In this study, we demonstrate that Bayes' theorem can be used to classify pathological severity with some certainty when only a small number of glomeruli are obtained during a single kidney biopsy. According to the Japanese guidelines, ≥ 10 glomeruli are required to accurately determine the pathological severity of the disease^8^. In the present study, the reliability of previous determinations using ≥ 10 glomeruli was as high as 91% (74–98), supporting the validity of the diagnostic criterion for 10 glomeruli. However, even in cases where the number of glomeruli drops below 10, such as if the total glomerular count was 6 and the number of lesions was 0, Grade 1 could be determined with very high probability which would be 96%. Thus, even though the number of glomeruli collected was ≤ 9, the probability would be very high like above case.

In preceding analyses, the prior distribution approximated the actual distribution of 99 cases using the beta distribution. The effect of the approximation owing to the insufficient number of cases is discussed by comparing the results of the distribution without prior information to the results of the prior distribution for all 99 cases.

When the actual distribution was used as the prior distribution, the reliability of the results was 86% (69–96) at the median. When the actual distribution was approximated by the beta distribution, the reliability was 87% (68–97). The reliability of the results was slightly lower (79% [62–92]) when all cases were equally distributed assuming no prior information. Therefore, it is necessary to increase the number of cases in the future to determine a reliable prior distribution.

In addition to the number of glomeruli, pathological classification using the lumped system in Japan seems to be influenced by a combination of the number of glomeruli and percentage of glomeruli showing disease in the total glomerular count. The Oxford classification required ≥ 8 glomeruli, whereas the Japanese classification required ≥ 10. This is because there was no significant difference in pathological severity observed among the three groups when divided into 8–12, 13–17, and ≥ 18 glomeruli^11^. In the posttest probability judgment (based on Bayes' theorem), accuracy of the diagnostic classification was indicated by a specific numerical value—referred to as the posterior probability—providing more detailed information about each case. This was a useful indicator in actual clinical practice, as there are several lumped systems for histological staging of IgA nephropathy that are also influenced by the number of glomeruli^22^.

This study has some limitations. First, we did not compare the Oxford and Japanese pathological classifications for IgA nephropathy, as they are different systems of classification. Second, due to the study design, cross-sectional and longitudinal studies are needed to clarify the pathological and clinical prognoses. Additionally, the original sample was from a single center, and the larger sample size and longitudinal study may be needed.

## Conclusion

The total number of collected glomeruli influenced the lumped IgA pathological classification system of the Japanese Society of Nephrology. IgA nephropathy could be diagnosed if only a few glomeruli were obtained by renal biopsy; in such cases, using Bayes’ theorem for probabilistic analysis would help to apply and interpret the Japanese Society of Nephrology IgA pathological classification.

## Data Availability

The datasets used and/or analyzed during the present study are available from the corresponding author upon reasonable request.
